# Is there a relationship between preoperative cytological diagnosis and evolution in patients with differentiated thyroid carcinoma? A retrospective study

**DOI:** 10.20945/2359-3997000000458

**Published:** 2022-04-19

**Authors:** Renato Colenci, Marcos Ferreira Minicucci, Carlos Segundo Paiva Soares, Cristiano Claudino de Oliveira, Mariângela Esther de Alencar Marques, José Vicente Tagliarini, Gláucia Maria Ferreira da Silva Mazeto

**Affiliations:** 1 Universidade Estadual Paulista Faculdade de Medicina de Botucatu Departamento de Clínica Médica Botucatu SP Brasil Departamento de Clínica Médica, Faculdade de Medicina de Botucatu, Universidade Estadual Paulista (Unesp), Botucatu, SP, Brasil; 2 Universidade Estadual Paulista Faculdade de Medicina de Botucatu Departamento de Oftalmologia Botucatu SP Brasil Departamento de Oftalmologia, Otorrinolaringologia e Cirurgia de Cabeça e Pescoço, Faculdade de Medicina de Botucatu, Universidade Estadual Paulista (Unesp), Botucatu, SP, Brasil; 3 Universidade Estadual Paulista Faculdade de Medicina de Botucatu Departamento de Patologia Botucatu SP Brasil Departamento de Patologia, Faculdade de Medicina de Botucatu, Universidade Estadual Paulista (Unesp), Botucatu, SP, Brasil

**Keywords:** Fine-needle biopsy, cytology, thyroid neoplasms, prognosis

## Abstract

**Objective:**

Cytological analysis and Bethesda classification of thyroid nodules is the standard method of diagnosing differentiated thyroid carcinoma (DTC). However, even for nodules with a non-malignant cytological diagnosis, there is a not insignificant risk of cancer. There are doubts whether this lack of certainty would influence patient prognosis. Our aim was to compare patients with DTC, classified according to the preoperative cytological diagnosis, regarding their evolution.

**Subjects and methods:**

A retrospective study was carried out with 108 DTC patients submitted to total thyroidectomy (TT) between 2009 and 2015, divided into three groups according to preoperative cytological diagnosis (Bethesda classification): classes I/II, III/IV, and V/VI. Groups were compared for evolution considering response to treatment at last evaluation as well as time disease free. Statistical analysis used ANOVA, chi squared, and Kaplan-Meier curves with p<0.05 considered significant.

**Results:**

Groups differed for time between nodule puncture and TT [in months; V/VI (2.35 ± 2.48) < III/IV (7.32 ± 6.34) < I/II (13.36 ± 8.9); p < 0.0001]. There was no significant difference between groups for evolution at final evaluation (disease free status; classes I/II: 71.4%; classes III/IV: 60%; classes V/VI: 66.6%; p = 0.7433), as well as time disease free (in months; classes I/II: 34.57 ± 25.82; classes III/IV: 38.04 ± 26.66; classes V/VI: 30.84 ± 26.34; p = 0.3841).

**Conclusions:**

DTC patients classified according to preoperative cytological diagnosis did not differ for evolution. Although patients with non-malignant cytological diagnoses were submitted to TT later, this did not affect the evolution of the cases.

## INTRODUCTION

Of all tumours which affect the endocrine system, thyroid cancer is the commonest malignant neoplasia with incidence rates increasing worldwide ( 1 , 2 ). Tumors that derive from follicular cells can be categorised into five histological groups: papillary carcinoma and its variants, follicular carcinoma and its variants, oncocytic carcinoma (from Hürthle cells), poorly differentiated carcinoma and undifferentiated carcinoma ( 3 ). The first three groups are generically classified as differentiated thyroid carcinomas (DTC), which correspond to around 90% of malignant thyroid neoplasms, mostly manifesting by the presence of nodules.

Fine needle aspiration biopsy (FNAB), with aspirated material analysis, is the standard method for evaluating thyroid nodules and diagnosing DTC, given its high rates of sensitivity (65% to 98%) and specificity (72% to 100%) ( 4 ), as well as being cost effective and having a low incidence of complications. Cytological diagnosis has been standardized since 2009, with the advent of the Bethesda classification ( 4 , 5 ), which indicates the therapeutic approach to these lesions.

Although DTC prognosis is generally good, some cases present in a more aggressive form or evolve with persistent disease during follow-up. In fact, 1%-30% of cases evolve to death due to neoplasia and up to 55% present disease persistence or recurrence ( 5 ), which can considerably impact patient quality of life and treatment costs ( 6 ). It is therefore imperative that cases are adequately evaluated for prognostic factors in order to consider how aggressive initial treatment and follow-up should be. In this sense, staging according to the American Joint Committee on Cancer (AJCC/TNM) ( 7 ) has been used to evaluate risk of death while the staging system proposed by the American Thyroid Association (ATA) ( 5 ) has been shown useful in predicting risk of disease recurrence. Both systems are used after initial therapeutic approach when definitive DTC diagnosis has already been established. Thus, markers that could also be used in the preoperative phase of these tumours and could help anticipate behaviour and more appropriate treatment planning are still needed.

In this context, some authors have reported that preoperative cytological diagnosis could be one more factor in DTC prognosis ( 8 – 13 ). However, despite the relevance of Bethesda classification in thyroid neoplasia diagnosis, its prognostic role is still controversial ( 14 , 15 ). Some of the factors responsible for this lack of consensus could be the lack of adherence to cytological classification standardization, which still persists, and a lack of uniformity in outcome assessment. In the latter case, ATA has proposed a dynamic classification system for response to initial treatment ( 5 ), which has shown promise in evaluating patient status during follow-up ( 16 , 17 ), and which could represent an adequate form of standardization for outcomes from different studies, in a similar way to Bethesda classification for cytological diagnoses ( 4 ).

The objective of this work was to compare patients with DTC classified according to preoperative cytological diagnosis as to whether or not they were free of the disease at last evaluation, according to the dynamic classification system, as well as the length of time free of the disease.

## MATERIALS AND METHODS

This retrospective observational study compared DTC patients according to their preoperative cytological diagnosis and evolution. Approval was obtained from our Research Ethics Committee before the start of research activities (CAAE n° 71719317.0.0000.5411).

### Study population and selection criteria

Sample size was calculated according to Lima and cols. ( 12 ), in which excellent response rates ( 5 ) of 92.1% and 74.5% were seen in Bethesda categories II and V/VI, respectively. Considering a test power of 80% and an alpha of 0.017 (Bonferroni correction for comparison between the three cytological groups: I/II, III/IV, and V/VI), minimum sample size was found to be 104 patients.

We initially evaluated 404 cases with anatomopathological DTC diagnosis followed in a specialised outpatient clinic at a tertiary hospital using a specific protocol ( 18 ). Succinctly, at the time of interest in this study, the local standard treatment for all cases was total thyroidectomy (TT) followed by radioiodine (^131^I) therapy (RIT). Lymphadenectomy was performed when suspected lymph node was detected on palpation and/or cervical ultrasonography and the presence of metastasis confirmed by FNAB, or when suspicious lymph node was found intraoperatively (therapeutical, and non-prophylactic lymphadenectomy). Patients were evaluated 3 months after TT with measurements of serum thyrotropin (TSH), antithyroglobulin antibody (TgAb), and thyroglobulin (Tg) stimulated by endogenous TSH (STg), and with whole-body scan (WBS) and ultrasound (US) cervical. Thereafter, patients underwent RIT with WBS approximately 5 days after the procedure. The RIT was scaled according to the initial extent of the disease, such that the doses administered were: 30 to 100 mCi (3.70 GBq) of ^131^I for low-risk cases; 150 mCi (5.55 GBq) for intermediate risk cases; 200 mCi (7.40GBq) for patients with advanced disease (T4 and/or M1 cases). Reassessments were performed every 4 to 6 months, through clinical examination, serum TSH, TgAb, and Tg measurements, and cervical US. One year after RIT, WBS and STg were still reassessed. In suspected persistent/recurrent neoplasia, additional imaging tests such as computed tomography, magnetic resonance, and positron emission computed tomography were requested. If necessary, cytological or histological exams were still performed.

**Figure 1:**
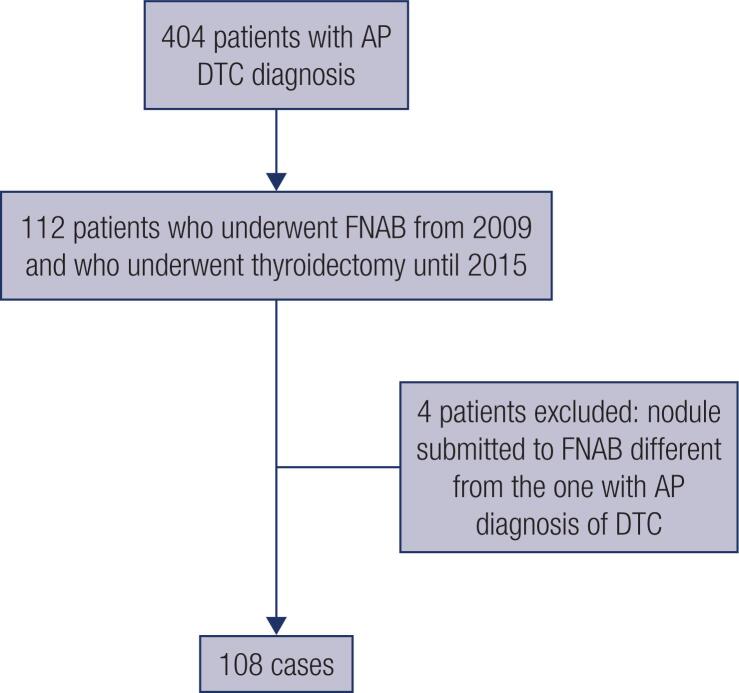
Flowchart illustrating the selection process of the patients. AP: anatomopathological; DTC: differentiated thyroid carcinoma; FNAB: Fine needle aspiration biopsy.

We included patients from 2009 who had been submitted to FNAB prior to surgery with cytological diagnosis classified according to Bethesda classification ( 4 ), and having undergone TT up to 2015, with a minimum postoperative follow-up of 24 months. Cases in which the punctured nodule differed from the nodule with a histological diagnosis of DTC were excluded. Thus 108 patients were effectively studied ( Figure 1 ).

### Evaluated variables and patient grouping

The main variable of interest in this study was the cytological diagnosis of the material obtained by FNAB from thyroid nodular lesions according to Bethesda classification ( 4 ). FNAB was indicated in the host hospital of the study, and during the specified data collection period, for nodules larger than 1cm or if smaller with suspicious characteristics from US (e.g. microcalcifications, blurred margins). In cases of multiple thyroid nodules, the dominant ones and those with suspect characteristics were punctured. All FNAB were guided by US, with a 23-gauge (25 x 6 mm) needle coupled to a 10 mL syringe. The aspirated material was stained with Giemsa and Papanicolau and slides were evaluated by two experienced cytologists and classified according to the Bethesda system ( 4 ). Cases were divided into three groups: classes I/II (non-diagnostic or unsatisfactory sample/benign), III/IV (atypia or follicular lesion of undetermined significance/suspicion or compatible with follicular neoplasm), and V/VI (suspected malignancy/malignant). The cytological classes were grouped to allow a statistical analysis with greater power, due to the sample number present in each class.

Other variables were also studied, including: age at time of surgery (years); gender (male; female); time between FNAB and surgery (in months); anatomopathological data (histological type, tumour diameter, multicentricity, lymphocytic thyroiditis, lymph nodal metastases, tumour capsule presence, tumour capsule invasion, vascular invasion, perineural invasion, and soft tissue invasion); staging for risk of death, according to the 7^th^ edition of the AJCC classification system/TNM ( 19 ), and for ATA recurrence risk (low, intermediate, or high) ( 5 ); first detectable STg [ng/mL; considered detectable if > 0,2 ng/mL; chemiluminescence method (DPC, Los Angeles, CA, USA)]; first detectable TgAb [considered detectable if > 40 UI/mL; chemiluminescence method (Immulite 2000, Siemens, Llanberis, Gwynedd, United Kingdom)]; result of the first WBS (negative uptake, positive cervical, and/or at distance uptake); first and cumulative doses of RIT (in mCi); presence and localization of metastases; and follow-up time (in months). Histopathological diagnosis of thyroid neoplasms was performed as previously described ( 20 ). Follow-up time, in months, was considered as the interval between TT and last consultation date.

### Evaluated outcomes

The main evaluated outcomes were the patient *status* at the last evaluation, classified as disease-free (DF) or not, and the DF survival time. The patient *status* was classified considering the response to initial treatment evaluated at the final consultation. According to the latest ATA guidelines, there are four responses: excellent, biochemically incomplete, structurally incomplete, and indeterminate ( 5 ). Patients were considered DF when having an excellent response, and non-DF (NDF) when having persistent/recurrent DTC (incomplete biochemical, structural, or indeterminate response) at that time. The DF survival time, in months, was defined as the time between TT and the date of eventual persistence/recurrence or of final consultation. We also calculated the relative DF survival time considering the percentage of time that patients were found free of the disease in relation to total follow-up time.

### Statistics

Data were entered into an Excel® (Microsoft Corporation, USA) spreadsheet and submitted to statistical analysis using SPSS/Windows® (version 21) and SAS® (version 9.4). Studied variables were described as means and standard deviations, and that analysis between groups and variables was by using ANOVA, followed by the Tukey test for multiple comparisons in numeric variables with normal distribution. Numbers with asymmetric distribution were adjusted by a generalised linear model with gamma distribution. For categorical variables, the Chi squared or Exact Fisher tests were applied, when necessary, the test of proportions was performed when statistical differences were found between groups. Disease-free survival was analysed using Kaplan-Meier curves, in function of preoperative cytological diagnosis. Values of p < 0.05 were considered as significant.

## RESULTS

Out of the 108 DTC patients included in the study ( Figure 1 ), most were female (87%), with mean age and follow-up times [±standard deviation (SD)] of 49.62 (±15.6) years and 49.06 (±16.7) months, respectively. There were 69 cases with cytological diagnosis classes V/VI (63.9%), 25 classes III/IV (23.1%), and 14 classes I/II (13%) ( Table 1 ).

**Table 1:** Clinical, cytohistological, laboratorial, and evolution data of the 108 patients

General data (n = 108)		
Female †		94 (87.0)
Age (years) §		49.62 ± 15.6
Cytologic diagnosis (Bethesda category) †		
	I	3 (2.8)
	II	11 (10.2)
	III	12 (11.1)
	IV	13 (12.0)
	V	31 (28.7)
	VI	38 (35.2)
Time elapsed between FNAB and TT (months) §		4.95 ± 6.1
Lymphadenectomy †		61 (56.5)
Histologic subtype †		
	Follicular carcinoma, oncocytic variant	4 (3.7)
	Follicular carcinoma	3 (2.8)
	Papillary carcinoma, follicular variant	39 (36.1)
	Papillary carcinoma, classic variant	57 (52.8)
	Papillary carcinoma, oncocytic variant	4 (3.7)
	Papillary carcinoma, solid variant	1 (0.9)
Lymphocytic thyroiditis †		42 (38.9)
	Tumor diameter (cm) §	1.90 ± 1.5
	Multicentricity †	60 (55.6)
	Tumoral capsule †	25 (23.2)
	Tumoral capsule invasion †	13 (12.0)
	Vascular invasion †	25 (23.1)
	Perineural invasion †	4 (3.7)
	Soft tissues invasion †	20 (18.5)
	Nodal metastasis †	31 (28.7)
AJCC/TNM staging (7^th^ edition) †		
	I	73 (67.6)
	II	13 (12.0)
	III	14 (13.0)
	IV	8 (7.4)
Recurrence risk (ATA 2015) †		
	Low	59 (54.6)
	Intermediary	31 (28.7)
	High	18 (16.7)
First STg (ng/mL) §		19.10 ± 61.5
First detectable TgAb †		18 (16.7)
First post-operative WBS †		
	Negative uptake	11 (10.2)
	Positive cervical uptake	90 (83.3)
	Positive cervical and distance uptake	6 (5.6)
First RIT dose (mCi) §		145.36 ± 55.3
Cumulative RIT dose (mCi) §		152.76 ± 83.4
Metastasis †		
	Nodal	26 (24.1)
	Nodal and lung	2 (1.9)
	Nodal. lung and spleen	1 (0.9)
	Lung	2 (1.9)
Total time of follow up (months) §		49.06 ± 16.7
Deaths †		1 (0.9)
DF in the last evaluation †		71 (65.7)
DF along all the follow up period †		54 (50.0)
DF total time (months) * §		32.99 ± 26.3
DF % of time ** §		63.7 ± 43.2

† n (%);

§ means ± standard deviation.

* Total time of follow up: time between total thyroidectomy to last evaluation.

** % of time free of disease: percentage of time free of disease considering the total time of follow up. AJCC: American Joint Committee on Cancer; cm: centimeters; DF: disease-free; FNAB: fine needle aspiration biopsy; mCi: miliCurie; RIT: radioiodine therapy; STg: thyroglobulin stimulated by endogenous thyrotropin; TgAb: antithyroglobulin antibody; TT: total thyroidectomy; WBS: whole body scan.

The three groups differed for mean time (±DP) in months between FNAB and TT: 2.35 (±2.48) for classes V/VI, 7.32 (±6.34) for classes III/IV, and 13.36 (±8.9) for classes I/II (V/VI < III/IV < I/II; p < 0.0001). No significant differences were observed between groups (p > 0.05) for percentage of DF cases at final evaluation, or in relation to DF time and the other evaluated variables ( Table 2 ).

**Table 2:** Comparison between the groups of patients according to the cytologic diagnosis, considering the variables studied

		Cytologic group †		
Variables	I/II 14 (13)	III/IV 25 (23.1)	V/VI 69 (63.9)	p
Female †	11 (78.5)	20 (80)	63 (91.3)	0.2122
Age (years) §	49.93 ± 18.68	48.68 ± 16.42	49.9 ± 14.92	0.9439
Papillary carcinoma †	13 (92.8)	21 (84)	67 (97.1)	0.074
Lymphocytic thyroiditis †	5 (35.7)	13 (52)	24 (57.1)	0.3077
Tumor diameter (cm) §	1.98 ± 2.14	2.12 ± 1.81	1.81 ± 1.2	0.6292
Multicentricity †	8 (61.5)	12 (48)	40 (59.7)	0.5669
Tumoral capsule †	3 (21.43)	9 (36)	13 (18.8)	0.2161
Tumoral capsule invasion †	2 (14.3)	3 (12)	8 (11.6)	0.9609
Vascular invasion †	3 (21.4)	6 (24)	16 (23.2)	0.9834
Perineural invasion †	2 (14.3)	0 (0)	2 (2.9)	0.0645
Soft tissues invasion †	3 (21.4)	1 (4)	16 (23.2)	0.1019
Nodal metastasis †	3 (21.4)	4 (16)	24 (34.8)	0.1670
AJCC/TNM stage I or II †	12 (85.7)	21 (84)	53 (76.8)	0.6213
Low risk of recurrence †	9 (64.2)	17 (68)	33 (47.8)	0.3002
First detectable STg §	10 (83.3)	18 (85.7)	45 (77.5)	0.6955
First detectable TgAb †	2 (15.3)	4 (16)	12 (17.4)	0.9767
First post-operative WBS with positive cervical uptake †	11 (78.5)	18 (72)	62 (89.8)	0.2703
First RIT dose (mCi) §	171.43 ± 80.18	134 ± 45	144.18 ± 51.81	0.1226
Cumulative RIT dose (mCi) §	171.43 ± 80,18	134 ± 45	155.78 ± 93.74	0.1626
Distant metastasis †	1 (7.1)	0 (0)	4 (5.8)	0.4433
Time between FNAB and TT (months) §	13.36 ± 8.9 c	7.32 ± 6.34 b	2.35 ± 2.48 a	**<0.0001**
Total time of follow up (months) §	51.78 ± 17.73	50.44 ± 16.74	48.01 ± 16.64	0.6699
DF in the last evaluation †	10 (71.4)	15 (60)	46 (66.7)	0.7433
DF total time (months) §	34.57 ± 25.82	38.04 ± 26.66	30.84 ± 26.34	0.3841
DF % of time considering the total time of follow up §	65.53 ± 41.83	70.15 ± 43.72	61.12 ± 43.68	0.6485

† n (%);

§ means ± standard deviation;

* p-value obtained through chi-square test for the categorical variables, ANOVA for the numeric variables with normal distribution and generalized linear model with gamma distribution for the numeric variables with asymmetric distribution;

a, b, c difference in proportion test between the groups compared by columns (c>b>a). Significance: p<0.05. AJCC: American Joint Committee on Cancer; DF: disease-free; FNAB: fine needle aspiration biopsy; mCi: miliCurie; RIT: radioiodine therapy; STg: thyroglobulin stimulated by endogenous thyrotropin; TgAb: antithyroglobulin antibody; TT: total thyroidectomy; WBS: whole body scan.

**Figure 2:**
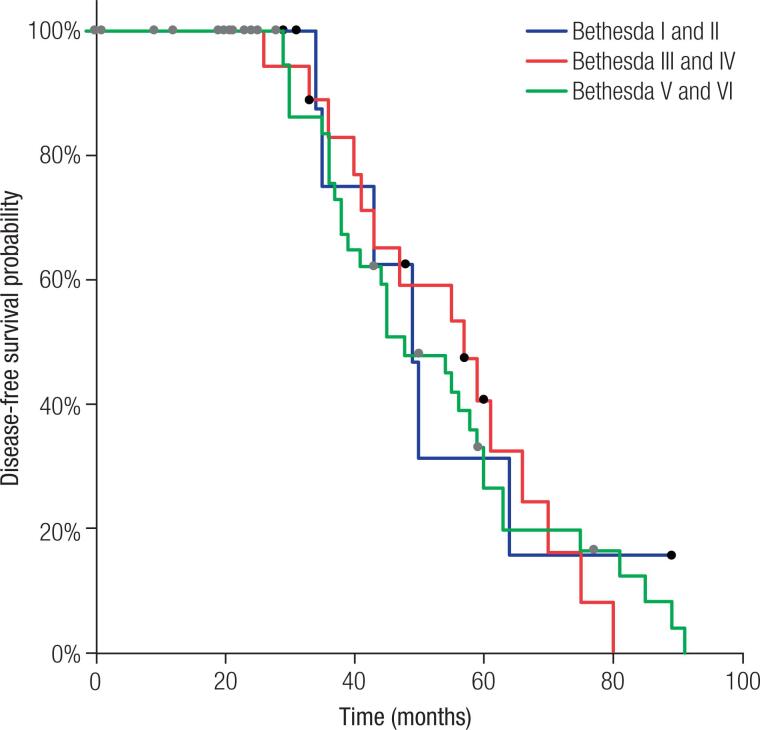
Kaplan-Meier curves for disease-free survival analysis, considering the pre-operative cytologic diagnosis (Log-Rank p = 0.9048; Wilcoxon p = 0.7789).

According to the Kaplan-Meier curves, the three groups also did not differ for disease-free survival time ( Figure 2 ; Log-Rank p = 0.9048; Wilcoxon p = 0.7789).

Considering the outcome status of the disease at last evaluation ( Table 3 ), seventy one patients (65.7%) were found DF and 37 (34.3%) NDF. The NDF patients presented a lower mean age [43.97 ± 19.96 *versus* ( *vs)* 52.56 ± 11.97 years; p = 0.0062]. There was an association between the outcome and the presence of lymph node metastases (56.7% in NDF *vs* 14% in DF; p < 0.0001), angiolymphatic invasion (35.1% in NDF *vs* 16.9% in DF; p = 0.033), soft tissue invasion (35.1% in NDF *vs* 9.8% in DF; p = 0.0013), and distant metastases (13.5% in NDF and no cases in DF; p = 0.0015). The NDF group still showed a higher percentage of cases at high recurrence risk (35.1 *vs* 7%; p < 0.001), and with first detectable STg (100% vs 70.4%; p = 0.0009), and received higher both initial (172.93 ± 70.30 *vs* 130.99 ± 39.7 mCi; p < 0.0001) and cumulative (194.55 ± 122.35 *vs* 130.99 ± 39.7 mCi; p < 0.0001) doses of ^131^I. On the other hand, a higher percentage of DF cases presented a lower recurrence risk (64.7 *vs* 35.1%; p < 0.002). The DF group also presented higher percentage of cases with lymphocytic thyroiditis (46.4 *vs* 24.3%; p = 0.025), more time between FNAB and TT (5.63 ± 6.69 *vs* 3.68 ± 4.75 months; p < 0.0001), total time in which patients were found free of the disease (46.35 ± 19.91 in DF *vs* 7.35 ± 15.95 months in NDF; p < 0.0001), and percentage of time free of the disease in relation to total follow-up time (90.2 ± 21.1 in DF *vs* 13.2 ± 26.2 months in NDF; p < 0.0001). Both groups did not differ in relation to percentage of cases in each cytological diagnosis class or in the remaining evaluated variables (p > 0.05).

**Table 3:** Comparison between patients free of disease and non-free of disease in the last evaluation, considering the clinical-epidemiologic variables

		Status in the last evaluation †		p
		Disease free 71 (65.7)	Non-disease free 37 (34.3)	
Female †		61 (85.9)	33 (89.1)	0.6307
Age (years) §		52.56 ± 11.97	43.97 ± 19.96	**0.0062**
Cytologic diagnosis (Bethesda) †				
	I / II	10 (14)	4 (10.8)	0.618
	III / IV	15 (21.1)	10 (37.0)	0.501
	V / VI	46 (64.7)	23 (62.1)	0.788
Papillary carcinoma †		68 (95.7)	33 (89.1)	0.1871
Lymphocytic thyroiditis †		33 (46.4)	9 (24.3)	**0.025**
Tumor diameter (cm) §		1.86 ± 1.62	1.99 ± 1.22	0.6254
Multicentricity †		39 (54.9)	21 (56.7)	0.8561
Tumoral capsule †		14 (19.7)	11 (29.7)	0.2417
Tumoral capsule invasion †		8 (11.2)	5 (13.5)	0.7335
Vascular invasion †		12 (16.9)	13 (35.1)	**0.033**
Perineural invasion †		1 (1.4)	3 (8.1)	0.0802
Soft tissue invasion †		7 (9.8)	13 (35.1)	**0.0013**
Nodal metastasis †		10 (14)	21 (56.7)	**<0.0001**
AJCC/TNM stage I or II †		57 (80.2)	29 (78.3)	0.8157
Recurrence risk †				
	Low	46 (64.7)	13 (35.1)	**0.002**
	Intermediary	20 (28.1)	11 (29.7)	0.866
	High	5 (7)	13 (35.1)	**0.001**
First detectable STg §		43 (70.4)	30 (100)	**0.0009**
First detectable TgAb †		10 (14)	8 (22.2)	0.2877
First post-operative WBS with positive cervical uptake †		64 (90.1)	29 (87.8)	0.7398
First RIT dose (mCi) §		130.99 ± 39.7	172.93 ± 70.3	**<0.0001**
Cumulative RIT dose (mCi) §		130.99 ± 39.7	194.55 ± 122.35	**<0.0001**
Distant metastasis †		0 (0.0)	5 (13.5)	**0.0015**
Time between FNAB and TT (months) §		5.63 ± 6.69	3.68 ± 4.75	**<0.0001**
Total time (months) of follow up §		50.75 ± 17.5	45.84 ± 14.75	0.1291
DF Total time (months) §		46.35 ± 19.91	7.35 ± 15.95	**<0.0001**
DF % of time considering the total time of follow up §		90.2 ± 21.1	13.2 ± 26.2	**<0.0001**

† n (%);

§ means ± standard deviation;

* p-value obtained through chi-square test for the categorical variables, ANOVA for the numeric variables with normal distribution and generalized linear model with gamma distribution for the numeric variables with asymmetric distribution. Significance: p < 0.05. AJCC: American Joint Committee on Cancer; DF: disease-free; FNAB: fine needle aspiration biopsy; mCi: miliCurie; RIT: radioiodine therapy; STg: thyroglobulin stimulated by endogenous thyrotropin; TgAb: antithyroglobulin antibody; TT: total thyroidectomy; WBS: whole body scan.

## DISCUSSION

In this study, we observed that the cytological diagnosis classes V/VI were associated with much earlier surgery, without however, impacting DTC patients as to DF outcome at last evaluation or in disease-free survival time.

There is still great controversy about the impact of preoperative cytological diagnosis on patient evolution. In this study, which used dynamic risk classification recommended by the latest ATA guidelines ( 5 ), no association was found between preoperative cytological diagnosis and patient clinical status at final evaluation, differing from that reported by other authors ( 11 ). Also, no association was found between disease-free survival time and cytological diagnosis, corroborating the findings of some studies ( 9 , 14 ), but disagreeing with others ( 11 , 13 ), who reported longer time in patients with a cytology showing lower probability of malignancy. As previously mentioned, the non-standardization of cytological diagnosis methods and of outcome evaluation could contribute to these divergences. In this study, we used two well-known systems: Bethesda Classification ( 4 ) and the ATA dynamic classification system of response to initial therapy ( 5 ). Certainly, the use of these tools will greatly facilitate multicentre communication, as well as providing greater agreement in application of actions.

We found an association between suspected or malignant cytological diagnosis (classes V/VI) and earliest surgical intervention, corroborating García-Pascual and cols. ( 14 ). In fact, preoperative cytological diagnosis, through FNAB, could impact the speed, and perhaps the aggressiveness of surgical approach. However, the impact of this diagnosis on patient evolution is still controversial. At first, the earlier approach to neoplastic diseases would appear associated with better case evolution. However, as already reported by other authors ( 15 ), early DTC diagnosis by FNAB, although reducing the time between nodule detection and thyroidectomy, does not interfere in patient evolution. In fact, in the present study, patients considered NDF at the last assessment were operated on earlier than those considered to be DF. In this context, there is a growing movement in developed countries for performing active follow-up for patients with < 1.5 cm sized malignant nodules (cytologies V/VI) without surgical intervention ( 21 , 22 ). Thus, serial follow-up is suggested every 6 to 12 months, with imaging evaluation by an experienced professional, preferably the same at all evaluations. Under the conditions reported by these authors, few tumours presented growth greater than 3 mm in 5 years, with this amount of tumour growth the parameter for surgical intervention ( 21 , 22 ). However, such conduct does not yet apply at all the centers, given the limitations for achieving serial follow-up under the proposed conditions. On the other hand, this could be a longer term alternative for reducing anaesthetic-surgical risks and unnecessary expenses, as it could provide an estimated 4 fold decrease in costs by performing active follow-up ( 21 ).

Our study did not reveal an association between cytological diagnosis and recurrence risk, corroborating results from other authors ( 12 ). However, a recent large study reported an association between both ( 10 ), guiding their conclusions towards a relationship between cytology and findings that could suggest a higher risk of recurrences, such as histological subtype and the presence of local recurrence and distant metastases. As the criteria to evaluate recurrence risk were different from the ones in our study ( 5 ), the comparison between both becomes difficult. Despite there being no association with cytological diagnosis in this study, risk of recurrence classified as high or low was associated with disease status at last evaluation, in accordance with the description of other authors ( 17 , 23 – 25 ). This association was not seen in patients classified as intermediate risk of recurrence.

Some studies have reported associations between cytological diagnoses V/VI (suspected malignancy/malignant) and highly aggressive anatomopathological characteristics such as the presence of extrathyroidal and angiolymphatic invasion and lymph node metastases ( 8 – 10 , 12 ), a fact not seen in our study, in which none of these anatomopathological parameters were associated with cytological diagnoses. In association analysis for disease outcome “status at final evaluation”, the presence of lymph node metastases, angiolymphatic invasion, perineural invasion, and the presence of lymphocytic thyroiditis were associated to the outcome, ratifying these as evolution markers ( 26 ), considered here in light of ATA criteria ( 5 ) for evaluating response to treatment. With the exception of lymphocytic thyroiditis, these parameters have been considered together in evaluating ATA recurrence risk ( 5 ). Some authors have reported a positive impact of the presence of chronic thyroiditis on the aggressiveness and evolution of DTC, particularly regarding the tumor dimensions and invasiveness, and the occurrence of metastases ( 27 ). However, the value of the presence of lymphocytic thyroiditis as a prognosis marker for DTC evolution is still not totally clear ( 28 ). Reasons for this controversy could include the diversity of criteria for diagnosing thyroiditis. The first question that arises is whether the DTC arose in the context of pre-existing autoimmune thyroiditis, with positive serum antibodies, or whether the thyroiditis was diagnosed during the pathological examination of the neoplasm. Are these histological findings the expression of tissue response to the tumor, with the potential to eventually limit tumor progression? Finally, this subject lacks further studies properly designed to clarify the still doubtful points.

In this study, the 7^th^ edition of AJCC/TNM ( 19 ) was considered, once that it was the parameter in force at the time of data collection. No association was seen between cytological diagnosis and risk of death, similar to descriptions by other authors ( 12 , 14 ), but different to the findings of VanderLaan and cols., who found an association between Bethesda cytology Class VI and the more advanced TNM stages ( 8 ). AJCC/TNM staging was also not associated to disease status in final evaluation, reinforcing the concept that it does not have prognostic value regarding therapeutic response or disease persistence, restricting itself as just a predictor of mortality from neoplasia ( 29 ). In this study, only one patient died due to DTC, who was also classified as AJCC/TNM Stage IV. A worrying finding was that 13% of the cases of DTC had a cytological diagnosis I/II. However, there are studies with similar designs to this one reporting even higher percentages (20.7 to 32.7%) of these cytological classes ( 12 , 14 ). The truth is that the rates of false-negative FNA have been very variable. For example, a robust study of 1369 patients and 2010 cytologically benign nodules, of which 325 were thyroidectomized (23.7%), reported percentages of just over 1% ( 30 ).

In this study, comparing the DF and NDF groups, other interesting results were observed regarding age, first STg, RIT, distant metastasis and disease-free time. The NDF group was younger at the time of TT, which was unexpected as older ages have been associated with a worse prognosis ( 31 , 32 ). Perhaps the reason for this difference between the results is due to the strict criteria used in the present study, in which only cases with excellent responses in the last assessment were classified as DF ( 5 ). The NDF group also had higher percentages of cases with detectable first STg. The prognostic value of STg, measured after TT and before RIT, both in the short and long term, has been reported by several authors ( 33 , 34 ). However, the groups classified according to the preoperative cytological class did not differ regarding this parameter, minimizing its influence on the evaluated outcomes. Regarding RIT, the NDF group received higher doses, which was expected since it had a higher percentage of cases at high risk of recurrence. Nevertheless, again, the groups classified according to cytological diagnosis did not differ regarding this parameter. Also, as expected, the NDF group had higher percentages of cases with distant metastases and remained for a shorter time in the disease-free state.

This study has the limitation of being retrospective in character and reflects the experience of a single-center, with a modestly sized but rigorously selected sample, which was perhaps not sufficient enough to demonstrate all the statistical differences. However, it is worth mentioning the value of having contemplated the most up-to-date guidelines regarding the parameters for diagnosing and following up DTC, which have until now had little scrutiny in literature. In addition, our results allow us to contemplate new hypotheses and record the experience of our service.

Concluding, in this study DTC patients classified according to different preoperative cytological diagnoses did not differ with respect to evolution. Despite cases with non-suspicious or compatible with malignancy having undergone treatment later, this did not interfere with either case status at last evaluation or disease-free survival time.
